# Coordinate-Descent Adaptation over Hamiltonian Multi-Agent Networks

**DOI:** 10.3390/s21227732

**Published:** 2021-11-20

**Authors:** Azam Khalili, Vahid Vahidpour, Amir Rastegarnia, Ali Farzamnia, Kenneth Teo Tze Kin, Saeid Sanei

**Affiliations:** 1 Department of Electrical Engineering, Malayer University, Malayer 65719-95863, Iran; matiin2441@yahoo.com (A.K.); v.vahidpour@gmail.com (V.V.); amir_rastegar2003@yahoo.com (A.R.); 2 Faculty of Engineering, Universiti Malaysia Sabah, Kota Kinabalu 88400, Malaysia; kenteo@ums.edu.my; 3 Science and Technology, Nottingham Trent University, Clifton Lane, Nottingham NG11 8NS, UK; saeid.sanei@ntu.ac.uk

**Keywords:** adaptive estimation, coordinate-descent, distributed networks, incremental algorithm

## Abstract

The incremental least-mean-square (ILMS) algorithm is a useful method to perform distributed adaptation and learning in Hamiltonian networks. To implement the ILMS algorithm, each node needs to receive the local estimate of the previous node on the cycle path to update its own local estimate. However, in some practical situations, perfect data exchange may not be possible among the nodes. In this paper, we develop a new version of ILMS algorithm, wherein in its adaptation step, only a random subset of the coordinates of update vector is available. We draw a comparison between the proposed coordinate-descent incremental LMS (CD-ILMS) algorithm and the ILMS algorithm in terms of convergence rate and computational complexity. Employing the energy conservation relation approach, we derive closed-form expressions to describe the learning curves in terms of excess mean-square-error (EMSE) and mean-square deviation (MSD). We show that, the CD-ILMS algorithm has the same steady-state error performance compared with the ILMS algorithm. However, the CD-ILMS algorithm has a faster convergence rate. Numerical examples are given to verify the efficiency of the CD-ILMS algorithm and the accuracy of theoretical analysis.

## 1. Introduction

Distributed processing over networks refers to the information extraction from streaming data collected at a group of spatially-dispersed and interconnected nodes. Distributed optimization problems of this kind appear in many applications, such as wireless sensor networks [1,2,3], multi-robot systems [4,5], smart grid programs [6,7,8,9], statistical learning over netwoks [10,11,12], and clustering [13,14]. Several classes of distributed optimization and estimation algorithms over multi-agent networks have been introduced in the literature. The consensus methods [15,16,17,18], incremental adaptive strategies [19,20,21,22,23,24], and diffusion networks [25,26,27,28,29] are among the most notable approaches.

In typical consensus strategies, each individual node collects noisy measurements over a period of time and performs a local processing task (e.g., calculates a local estimate). Then, the nodes collaborate through several iterations and share their information to achieve agreement. In the incremental strategies, a Hamilton cycle is established through the nodes where each node receives data from previous node on cycle path and sends the updated data to the next node. Such a mode of cooperation decreases the inter-node communication across the network and modifies the network independence. In diffusion strategies, information is spread through the network simultaneously and locally at all nodes and the task of distributed processing is performed. As in this mode of cooperation each node collaborates with its connected neighbors, the communication burden is higher than that of an incremental based strategy.

In this paper, we propose an incremental-based algorithm for solving the distributed estimation problem over a Hamilton network. One of the primary methods for solving this problem is the incremental least-mean-square (ILMS) algorithm reported in [19]. In this algorithm, the local calculated estimates are updated at each node by employing the LMS adaptation rule, without requiring any prior information about the data statistics. In order to implement the ILMS algorithm, it is assumed that at each time instant, the adaptation step has access to all entries of the approximate gradient vector—see expression (3) further ahead. In some practical situations (e.g., limited communication resources or missing data due to imperfect communication links) only a fraction of the approximate gradient vector is available for adaption. Thus, developing a new version of ILMS algorithm for networks with mentioned scenarios is of practical importance.

So far, different algorithms have been reported in the literature which solve the problem of distributed learning with partial information. In [30,31,32], a Bernoulli variable is used to model the step-size parameter, so that when the step-size is zero, not all entries of intermediate estimates are updated and the adaptation step is skipped for some. In [33,34,35,36,37,38], a different notion of partial diffusion of information is employed which relies on the exchange of a subset of entries of the weight estimate vectors themselves. In other words, it is assumed that each node shares only a fraction of its local information with its neighbors. Likewise, in [39], a situation is assumed in which some entries in the regression vector are missing at random due to incomplete information or censoring. In order to undo these changes, authors in [39] proposed an estimation scheme to estimate the underlying model parameters when the data are missing. Some other criteria have been proposed in the literature to motivate partial updating scheme. For instance, in [40], the periodic and sequential partial LMS updates are proposed, where the former scheme updates all filter coefficients every P-th iteration, with P>1, instead of every iteration. The later updates only a portion of coefficients, which are predetermined, at each iteration. In [41], the stochastic partial LMS algorithm is proposed that is a randomized version of sequential scheme where the coefficient subsets are selected in random instead of deterministic fashion. The works [42,43] utilize the concept of set-membership filtering to partially update the weight vector, where the updates occur only when the innovation obtained from the data exceeds a predetermined threshold. The works [12,44,45] employ Krylov subspace concept to partially update the weight vector that is based on dimensionality reduction policies. Some other techniques based on energy considerations limit the updates, e.g., [46]. In [47], a reduced complexity augmented complex least-mean-square algorithm is proposed by employing partial-update for improper complex signals. The algorithm involves selection of a fraction of coefficients at every iteration. In [48], the authors focus on diffusion learning mechanisms and study its mean-square error performance under a generalized coordinate-descent scheme where the adaptation step by each node involves only a random subset of the coordinate-descent gradient vector. None of the aforementioned works; however, rely on the adaptive incremental strategy. To address this issue, we combine two techniques (incremental mode of cooperation and coordinate-descent updates) to consolidate a new method, termed coordinate-descent ILMS (CD-ILMS), for distributed estimation problem.

The main contributions of this manuscript can be stated as follows:

(a) A CD-ILMS algorithm is proposed to simulate the situation where some entries in approximate gradient vectors are missing or to control the computational burden of the update estimate purposely;
  1. We examine the effect of random partial gradient information on the learning performance and convergence rate of ILMS algorithm for MSE cost function;
  2. A theoretical analysis of the performance of CD-ILMS algorithm is concluded under some typical simplifying assumptions and approximations, typical in adaptive systems and tend to achieve a performance level that matches well with practice;
  3. Stability conditions for CD-ILMS algorithm are derived both in mean and mean-square senses under certain statistical conditions. We find the necessary condition on step-size μ to guarantee the stability of CD-ILMS algorithms;
  4. Employing the energy conservation paradigm, we derive closed-form expressions to describe the learning curves in terms of excess mean-square-error (EMSE) and mean-square-deviation (MSD);
  5. We compare the CD-ILMS algorithm and regular full-update ILMS algorithm in terms of convergence rate and computational complexity. We find that although the CD-ILMS algorithm convergence to steady-state region takes longer, the overall computational complexity, i.e., savings in computation per iteration, does remain invariant.

The paper layout is organized as follows: In Section 2, we briefly explain the ILMS algorithm and formulate the CD-ILMS algorithm. In Section 3.1, we describe the system model and assumptions. The stability and performance analysis of CD-ILMS algorithm are analyzed both in mean and mean-square sense in Section 3.2 and Section 3.3, respectively. Section 3.4 and Section 3.5 investigate the transient and steady-state performance of CD-ILMS algorithm, respectively. In Section 4, we draw a comparison between ILMS and CD-ILMS algorithms on the basis of convergence rate and computational complexity. Performance evaluations are illustrated in Section 5. The paper is finally concluded in Section 6.

**Notation:** Throughout the paper, we adopt normal lowercase letters for scalars, bold lowercase letters for column vectors and bold uppercase letters for matrices, while I denotes an identity matrix of appropriate size. Superscript ${\left(.\right)}^{T}$ denotes transposition for real-valued vectors and matrices. The symbol $E\;\left[.\right]$ is the expectation operator, $\mathrm{tr}\left\{.\right\}$ represents the trace of its matrix argument, $\mathrm{vec}\left\{.\right\}$ vectorizes a matrix by stacking its columns on top of each other, and ⊗ is the standard Kronecker product.

## 2. Algorithm Description

**Figure 1:**
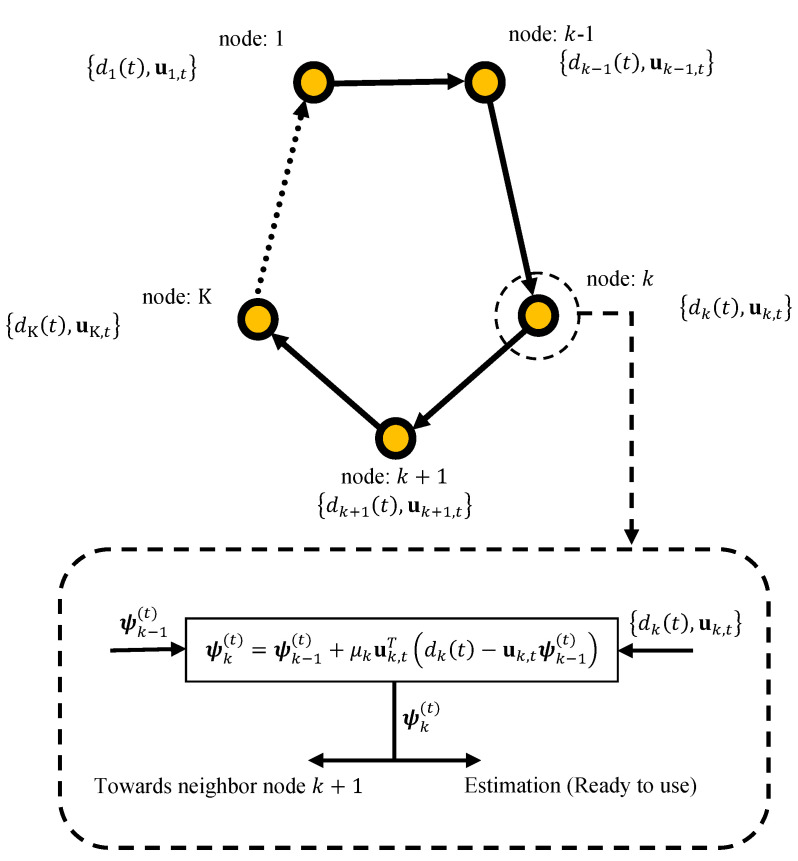
Data processing in an incremental network.

We consider a network with K nodes with incremental cooperation topology, as shown in Figure 1. The network is used to estimate the parameter $w^{o}\in R^{L\times 1}$, that minimize the following aggregate global cost function:

(1) $$\begin{array}{c} \min_{w}f^{g}\left(w\right)≜\sum_{k=1}^{K}f_{k}\left(w\right) \end{array}$$

where it is assumed that each individual cost function, $f_{k}\left(w\right):R^{L\times 1}\to R$ is ν-strongly convex and its gradients satisfy the δ-Lipschitz condition  [25]. These conditions are equivalent to requiring $f_{k}\left(w\right)$ to be twice-differentiable and Hessian matrices of the individual costs, $\nabla_{w}^{2}f_{k}\left(w\right)\in R^{L\times L}$, to be bounded as follows

(2) $$\begin{array}{c} 0<\nu I_{L}\leq \nabla_{w}^{2}f_{k}\left(w\right)\leq \delta I_{L} \end{array}$$

for some positive parameters $\left\{\nu ,\delta \right\}$, where ν<δ. Some cases of interest, e.g., Log-Loss regression or MSE [25,26], automatically satisfy the condition in (2).

### 2.1. Incremental Steepest-Descent Solution

By applying the steepest-descent method in conjunction with the incremental strategy to (1) gives the Incremental Steepest-Descent Solution as lists in Algorithm 1. Observe that in this implementation, each node *k* needs to receive the calculated estimate of $w^{o}$ at node k−1 (which is denoted by $\psi_{k-1}^{\left(t\right)}$). In (3), $w_{t}$ denotes a global estimation of $w^{o}$ at time instant *t*. Moreover, since the true gradient vectors, $\left\{\nabla_{w^{T}}f_{k}\left(\cdot \right)\right\}$, are not available beforehand in many practical applications, they have been replaced by approximates, $\left\{\hat{\nabla_{w^{T}}f_{k}}\left(\cdot \right)\right\}$.
**Algorithm 1.** Incremental Strategy [19].**Initialization:** start with $w_{0}=$ initial condition.**for** every time t≥1 **do**    set $\psi_{0}^{\left(t\right)}=w_{t-1}$    **for** nodes k=1,…,K **do**        receive $\psi_{k-1}^{\left(t\right)}$ from node k−1
$$\begin{array}{c} \;\psi_{k}^{\left(t\right)}=\psi_{k-1}^{\left(t\right)}-\mu_{k}\hat{\nabla_{w^{T}}f_{k}}\left(\psi_{k-1}^{\left(t\right)}\right) \end{array}$$    **end for**    set $w_{t}=\psi_{K}^{\left(t\right)}$**end for****End**

### 2.2. Coordinate-Descent Incremental Adaptation

Consider a case in which, due to some practical issues (e.g., missing data or limited computational and communication burden) only a subset of the approximate gradient vector can be updated as follows:

(3) $$\psi_{k}^{\left(t\right)}=\psi_{k-1}^{\left(t\right)}-\mu_{k}\hat{\nabla_{w^{T}}f_{k}}\left(\psi_{k-1}^{\left(t\right)}\right)$$

In order to handle such a situation, first a suitable model should be adopted. To this end, we follow a similar approach to [48] and define a diagonal random matrix $\Pi_{k,t}\in R^{L\times L}$ of node *k* at iteration *t* as

(4) $$\begin{array}{c} \Pi_{k,t}≜\mathrm{diag}\left\{\pi_{k,t}\left(1\right),\pi_{k,t}\left(2\right),\ldots ,\pi_{k,t}\left(L\right)\right\} \end{array}$$

where $\left\{\pi_{k,t}\left(l\right)\right\}$ are some Bernoulli random variables, that the randomness in the update varies across space, *k*, and over time, *t*. We have $\pi_{k,t}\left(l\right)=0$ or $\pi_{k,t}\left(l\right)=1$ with the following probability

(5) $$\begin{array}{c} \mathrm{Prob}\left(\pi_{k,t}\left(l\right)=0\right)=\pi_{k};\;0\leq \pi_{k}<1 \end{array}$$

where $\pi_{k,t}\left(l\right)=0$ means that *l*-th entry of $\hat{\nabla_{w^{T}}f_{k}}\left(\psi_{k-1}^{\left(t\right)}\right)$ is missing and *l*-th entry of $\psi_{k,t}$ in (3) is not updated. Multiplying the approximate gradient vector by $\Pi_{k,t}$, we replace $\hat{\nabla_{w^{T}}f_{k}}\left(\psi_{k-1}^{\left(t\right)}\right)$ by ${\hat{\nabla_{w^{T}}f_{k}}}^{\mathrm{miss}}\left(\psi_{k-1}^{\left(t\right)}\right)$ as

(6) $$\begin{array}{c} {\hat{\nabla_{w^{T}}f_{k}}}^{\mathrm{miss}}\left(\psi_{k-1}^{\left(t\right)}\right)=\Pi_{k,t}\times \hat{\nabla_{w^{T}}f_{k}}\left(\psi_{k-1}^{\left(t\right)}\right) \end{array}$$

## 3. Performance Analysis

### 3.1. Data Model and Assumptions

Consider MSE networks where at each time instant *t*, each node *k* is assumed to observe a scalar measurement $d_{k}\left(t\right)\in R$ and a 1×L row regression vector $u_{k,t}\in R^{1\times L}$. A linear regression model is used to describe the collected data at each individual node as follows:

(7) $$\begin{array}{c} d_{k}\left(t\right)=u_{k,t}w^{o}+v_{k}\left(t\right) \end{array}$$

where $v_{k}\left(t\right)$ is the noise process. The individual cost function, $f_{k}\left(w\right)$, that is associated with each node *k* is the MSE (quadratic) cost

(8) $$\begin{array}{c} f_{k}\left(w\right)=E\;\left[{\left|d_{k}\left(t\right)-u_{k,t}w\right|}^{2}\right] \end{array}$$

To proceed with analysis, we introduce some notation and assumptions. In our analysis, we assume that:

**Assumption** **1.** *(i) For all nodes k, and t≥1, the measurement noises $\left\{v_{k}\left(t\right)\right\}$ are all zero-mean, white, and independent from the input and desired signals, with variances $\sigma_{v,k}^{2}$;*

 *1.*  *The regression data $u_{k,t}$ for all nodes k, and all observation times t≥1, are zero-mean, white overt time and space with*
$$\begin{array}{c} E\;\left[u_{k,t}^{T}u_{\ell ,t}\right]≜R_{u,k}\delta_{k\ell }\delta_{st} \end{array}$$
  *where $0<R_{u,k}\in R^{L\times L}$, and $\delta_{k\ell }$ denotes the Kronecker delta, i.e., it is equal to one when k=ℓ and zero otherwise;*
 *2.*  *The step-sizes, $\mu_{k}$, are small enough so as to ignore the quadratic term in $\mu_{k}$.*

Let the notation $K_{t-1}$ represent the available information about the random processes $\psi_{k}^{\left(\tau \right)}$ and $\Pi_{k,\tau }$ at all agents k=1,2,…,K for τ≤t−1:

(9) $$\begin{array}{c} K_{t-1}≜\mathrm{filtration}\left\{\psi_{k}^{\left(\tau \right)},\Pi_{k,\tau }\right\} \end{array}$$

**Assumption** **2.** *For all k,ℓ,m,n, the indicator variables $\pi_{k,t}\left(m\right)$ and $\pi_{k,t}\left(n\right)$ are assumed to be independent of each other. Moreover, the random variables $\pi_{k,t}\left(m\right)$ and $K_{t-1}$ are independent of each other, for any iteration $w\in K_{t-1}$ and for all nodes k.*

Based on the adaptive implementation of incremental strategy [19], the adaptive distributed coordinate-descent incremental algorithm or distributed coordinate-descent incremental LMS (CD-ILMS) algorithm can be summarized in Algorithm 2. In this algorithm, each node updates the local estimate as

(10) $$\begin{array}{c} \psi_{k}^{\left(t\right)}=\psi_{k-1}^{\left(t\right)}+\mu_{k}\Pi_{k,t}u_{k,t}^{T}\left(d_{k}\left(t\right)-u_{k,t}\psi_{k-1}^{\left(t\right)}\right) \end{array}$$

**Algorithm 2.** Coordinate-Descent Incremental LMS (CD-ILMS) Strategy.**Initialization:** start with $w_{-1}=$ initial condition.**for** every time t≥0 **do**    set the fictitious boundary condition at $\psi_{0}^{\left(t\right)}\leftarrow w_{t-1}$.    **for** nodes k=1,…,K **do**        node *k* receives $\psi_{k-1}^{\left(t\right)}$ from its preceding neighbor        k−1,        node *k* performs:
$$\begin{array}{c} \;\psi_{k}^{\left(t\right)}=\psi_{k-1}^{\left(t\right)}+\mu_{k}\Pi_{k,t}u_{k,t}^{T}\left(d_{k}\left(t\right)-u_{k,t}\psi_{k-1}^{\left(t\right)}\right) \end{array}$$    **end for**    set $w_{t}\leftarrow \psi_{K}^{\left(t\right)}$
**end for**

**End**


### 3.2. Mean Stability Analysis

Now, the mean behavior of the proposed algorithm is analyzed. More specifically, we seek conditions that for sufficiently large *t* and for all *k*, the proposed algorithm is asymptotically unbiased. To proceed, the following local error signals are defined:

$$\begin{array}{c} {\tilde{\psi }}_{k}^{\left(t\right)}≜w^{o}-\psi_{k}^{\left(t\right)},\;{\tilde{w}}_{t}≜w^{o}-w_{t} \end{array}$$

By replacing $d_{k}$ from (7) into (10) and subtracting $w^{o}$ from both sides, gives

(11) $$\begin{array}{c} {\tilde{\psi }}_{k}^{\left(t\right)}=\left(I_{L}-\mu_{k}\Pi_{k,t}u_{k,t}^{T}u_{k,t}\right){\tilde{\psi }}_{k-1}^{\left(t\right)}-\mu_{k}\Pi_{k,t}u_{k,t}^{T}v_{k}\left(t\right) \end{array}$$

Taking statistical expectations of both sides of (11) together with using the items on Assumption 1, we obtain

(12) $$\begin{array}{c} E\;\left[{\tilde{\psi }}_{k}^{\left(t\right)}\right]=\left(I_{L}-\mu_{k}\left(1-\pi_{k}\right)R_{u,k}\right)E\;\left[{\tilde{\psi }}_{k-1}^{\left(t\right)}\right] \end{array}$$

Through iterating recursion (12) over *t* and setting k=K, we deduce that $E\;\left[{\tilde{\psi }}_{k}^{\left(t\right)}\right]$ evolves according to

(13) $$\begin{array}{cc} E\;\left[{\tilde{w}}_{t}\right] & =\left[\prod_{k=1}^{K}\left(I_{L}-\mu_{k}\left(1-\pi_{k}\right)R_{u,k}\right)\right]E\;\left[{\tilde{w}}_{t-1}\right]\\ \; & =\left[\prod_{k=1}^{K}Q_{k}\right]E\;\left[{\tilde{w}}_{t-1}\right]=QE\;\left[{\tilde{w}}_{t-1}\right] \end{array}$$

where $Q_{k}≜\left(I_{L}-\mu_{k}\left(1-\pi_{k}\right)R_{u,k}\right)$. Clearly, the mean weight-error vector for the CD-ILMS depends on the spectral radius of Q. The following proposition summarizes the required mean stability condition for the proposed algorithm.

**Proposition** **1** **(Mean Stability).** *Assume the data model (7) and Assumption 1 hold. Then, the CD-ILMS algorithm is asymptotically unbiased if, and only if, the step-size parameters $\left\{\mu_{k}\right\}$ satisfy the following condition:*

(14) $$0<\mu_{k}<\frac{2}{\left(1-\pi_{k}\right)\lambda_{\max}\left\{R_{u,k}\right\}}$$

*where $\lambda_{\max}$ denotes the largest eigenvalue of its argument.*

**Proof.**  The asymptotic unbiasedness of the CD-ILMS algorithm is guaranteed if, and only if, the matrix Q be stable, or equivalently, all its eigenvalues lie inside the unit disc, namely,

(15) $$\begin{array}{c} \rho \left(Q\right)<1 \end{array}$$

As the spectral radius of a matrix is always smaller than any induced norm of the same matrix [49] we have

(16) $$\begin{array}{cc} \rho \left(Q\right)\leq ∥Q∥\; & \leq ∥Q_{1}∥∥Q_{2}∥\ldots ∥Q_{K}∥\\ \; & \overset{*}{=}\rho \left(Q_{1}\right)\rho \left(Q_{2}\right)\ldots \rho \left(Q_{K}\right) \end{array}$$

where step $\left(*\right)$ is because every $Q_{k}$ is a symmetric matrix. This means that its spectral radius agrees with its 2-induced norm, so that $\left(*\right)$ is justified. Accordingly, to guarantee the constraint (15) for all *k*, it is enough to have $\rho \left(Q_{k}\right)\leq 1$, which is equivalent to

(17) $$\begin{array}{c} \left|1-\mu_{k}\left(1-\pi_{k}\right)\lambda_{\max}\left\{R_{u,k}\right\}\right|<1\;\forall \;k. \end{array}$$

Thus, the conclusion in (14) is verified. □

### 3.3. Mean-Square Performance

In this section, the mean-square performance of the error recursion (11) is examined. We start by equating the squared-weighted Euclidean norms of both sides of (11) and taking the expectations together with employing Assumption 1. After a straightforward calculation, we find the following weighted-variance relation:

(18) $$\begin{array}{cc} E\;\left[{∥{\tilde{\psi }}_{k}^{\left(t\right)}∥}_{\Sigma }^{2}\right]= & \;E\;\left[{∥{\tilde{\psi }}_{k-1}^{\left(t\right)}∥}_{\Sigma^{\prime }}^{2}\right]\\ \; & +\mu_{k}^{2}\sigma_{v,k}^{2}E\;\left[u_{k,t}\Pi_{k,t}\Sigma \Pi_{k,t}u_{k,t}^{T}\right] \end{array}$$

(19) $$\begin{array}{c} \Sigma^{\prime }=\left(I_{L}-\mu_{k}u_{k,t}^{T}u_{k,t}\Pi_{k,t}\right)\Sigma \left(I_{L}-\mu_{k}\Pi_{k,t}u_{k,t}^{T}u_{k,t}\right) \end{array}$$

for any arbitrary positive-definite weighting matrix Σ. Due to the assumption on $v_{k}\left(t\right)$, the expectations of the cross-terms involving $v_{k}\left(t\right)$ evaluate to zero. Note that items in Assumption 1 guarantee that ${\tilde{\psi }}_{k-1}^{\left(t\right)}$ is independent of $\Sigma^{\prime }$ and $u_{k,t}$. Thus, the expectation $E\;\left[{∥{\tilde{\psi }}_{k-1}^{\left(t\right)}∥}_{\Sigma^{\prime }}^{2}\right]$ can be rewritten as

(20) $$\begin{array}{c} E\;\left[{∥{\tilde{\psi }}_{k-1}^{\left(t\right)}∥}_{\Sigma^{\prime }}^{2}\right]=E\;\left[{∥{\tilde{\psi }}_{k-1}^{\left(t\right)}∥}_{E\;\left[\Sigma^{\prime }\right]}^{2}\right] \end{array}$$

By defining

(21) $$\begin{array}{cc} \sigma & ≜\mathrm{vec}\left\{\Sigma \right\} \end{array}$$

(22) $$\begin{array}{cc} \sigma^{\prime } & ≜\mathrm{vec}\left\{E\;\left[\Sigma^{\prime }\right]\right\} \end{array}$$

and employing (20)–(22), we can modify (18) to

(23) $$\begin{array}{cc} E\;\left[{∥{\tilde{\psi }}_{k}^{\left(t\right)}∥}_{\sigma }^{2}\right]= & \;E\;\left[{∥{\tilde{\psi }}_{k-1}^{\left(t\right)}∥}_{\sigma^{\prime }}^{2}\right]\\ \; & +\mu_{k}^{2}\sigma_{v,k}^{2}E\;\left[u_{k,t}\Pi_{k,t}\Sigma \Pi_{k,t}u_{k,t}^{T}\right] \end{array}$$

where $E\;\left[{∥{\tilde{\psi }}_{k}^{\left(t\right)}∥}_{\sigma }^{2}\right]$ and $E\;\left[{∥{\tilde{\psi }}_{k-1}^{\left(t\right)}∥}_{\sigma^{\prime }}^{2}\right]$ refer to the same quantities as $E\;\left[{∥{\tilde{\psi }}_{k-1}^{\left(t\right)}∥}_{\Sigma }^{2}\right]$ and $E\;\left[{∥{\tilde{\psi }}_{k-1}^{\left(t\right)}∥}_{\Sigma^{\prime }}^{2}\right]$, respectively.

Taking the vectorization operator of both sides of (19), and using (21), (22), Assumptions 1 and 2, together with employing the relationship between the Kronecker product and the vectorization operator (For any matrices $\left\{A,\Sigma ,B\right\}$ of compatible dimensions we have $\mathrm{vec}\left\{A\Sigma B\right\}=\left(B^{T}⊗A\right)\mathrm{vec}\left\{\Sigma \right\}$) we find that the weighting vectors $\left\{\sigma ,\sigma^{\prime }\right\}$ satisfy the following relation

(24) $$\begin{array}{c} \sigma^{\prime }=F_{k}\sigma \end{array}$$

where $F_{k}\in R^{L^{2}\times L^{2}}$ and given by

(25) $$\begin{array}{c} F_{k}≜E\;\left[\left(I_{L}-\mu_{k}u_{k,t}^{T}u_{k,t}\Pi_{k,t}\right)⊗\left(I_{L}-\mu_{k}\Pi_{k,t}u_{k,t}^{T}u_{k,t}\right)\right] \end{array}$$

Note that the vectorization commutes through expectation. Considering Assumption 1, we can approximate $F_{k}$ as follows

(26) $$\begin{array}{c} F_{k}\approx \left[\left(I_{L}-\mu_{k}\left(1-\pi_{k}\right)R_{u,k}\right)⊗\left(I_{L}-\mu_{k}\left(1-\pi_{k}\right)R_{u,k}^{T}\right)\right] \end{array}$$

To evaluate $E\;\left[u_{k,t}\Pi_{k,t}\Sigma \Pi_{k,t}u_{k,t}^{T}\right]$ we employ a useful property from matrix algebra (which relates the vectorization operator matrix to trace operator (For real matrices $\left\{A,B\right\}$, the trace of a product can be written as $\mathrm{tr}\left\{A^{T}B\right\}={\mathrm{vec}}^{T}\left\{B\right\}\mathrm{vec}\left\{A\right\}$) [50]) together with the fact that Σ is symmetric and deterministic, to obtain

(27) $$\begin{array}{cc} E\;\left[u_{k,t}\Pi_{k,t}\Sigma \Pi_{k,t}u_{k,t}^{T}\right] & =E\;\left[\mathrm{tr}\left\{\Sigma \Pi_{k,t}u_{k,t}^{T}u_{k,t}\Pi_{k,t}\right\}\right]\\ \; & ={\mathrm{vec}}^{T}\left\{E\;\left[\Pi_{k,t}u_{k,t}^{T}u_{k,t}\Pi_{k,t}\right]\right\}\sigma \\ \; & ={\mathrm{vec}}^{T}\left\{H_{k}\right\}\sigma \end{array}$$

where

(28) $$\begin{array}{cc} H_{k} & ≜E\;\left[\Pi_{k,t}u_{k,t}^{T}u_{k,t}\Pi_{k,t}\right] \end{array}$$

which, in the light of Assumptions 1 and 2, can be evaluated as

(29) $$\begin{array}{cc} H_{k} & =E\;\left[\Pi_{k,t}R_{u,k}\Pi_{k,t}\right] \end{array}$$

It follows by direct inspection that the entries of $H_{k}$ are given by

(30) $$\begin{array}{c} H_{k}\left(p,q\right)=\left\{\begin{array}{cc} {\left(1-\pi_{k}\right)}^{2}R_{u,k}\left(p,q\right), & p\neq q\\ \left(1-\pi_{k}\right)R_{u,k}\left(p,p\right), & p=q \end{array}\right. \end{array}$$

where

$$\begin{array}{c} E\;\left[\Pi_{k,t}\Pi_{\ell ,t}\right]=\left\{\begin{array}{cc} \left(1-\pi_{k}\right)\left(1-\pi_{\ell }\right), & k\neq \ell \\ \left(1-\pi_{k}\right), & k=\ell \end{array}\right. \end{array}$$

Substitution of (24) and (27) into (23) gives

(31) $$\begin{array}{cc} E\;\left[{∥{\tilde{\psi }}_{k}^{\left(t\right)}∥}_{\sigma }^{2}\right]= & \;E\;\left[{∥{\tilde{\psi }}_{k-1}^{\left(t\right)}∥}_{F_{k}\sigma }^{2}\right]+\mu_{k}^{2}\sigma_{v,k}^{2}{\mathrm{vec}}^{T}\left\{H_{k}\right\}\sigma \end{array}$$

In the following proposition, we summarize the required conditions that guarantee the mean-square convergence for the DC-ILMS algorithm.

**Proposition** **2** **(Mean-square Stability).** *Assume the data $\left\{d_{k}\left(t\right),u_{k,t}\right\}$ satisfy the model (7). Under Assumptions 1 and 2, the recursion of type (31) is stable and convergent if, and only if, $F_{k}$ given by (25) is stable. On the other hand, the stability of $F_{k}$ is achieved when $\left\{\mu_{k}\right\}$ are chosen, such that they satisfy (14).*

**Proof.**  Let A and B be two arbitrary matrices of compatible dimensions. Then, any eigenvalue of A⊗B is a product $\lambda_{i}\left(A\right)\times \lambda_{j}\left(B\right)$, where $\lambda_{i}\left(A\right)$ is an eigenvalue of A and $\lambda_{j}\left(B\right)$ is an eigenvalue of B [50]. Moreover, the sets of eigenvalues of A and $A^{T}$ are equal. Accordingly, using the expression (26), we have that $\rho \left(F_{k}\right)={\left[\rho \left(I_{L}-\mu_{k}\left(1-\pi_{l}\right)R_{u,k}\right)\right]}^{2}$. It follows that $F_{k}$ is stable if, and only if, $\left(I_{L}-\mu_{k}\left(1-\pi_{l}\right)R_{u,k}\right)$ is stable. Therefore, $\left\{\mu_{k}\right\}$ that guarantee the mean stability, ensure mean-square stability as well. Thus, the result (14) follows. □

### 3.4. Learning Curves

In this section, the variance relation (31) is employed to obtain a recursive equation that explains the transient behavior of the CD-ILMS algorithm. Since the weighting matrices, $\left\{\Sigma ,\Sigma^{\prime }\right\}$, can be node-dependent, we can replace $\left\{\sigma ,\sigma^{\prime }\right\}$ by $\left\{\sigma_{k},\sigma_{k}^{\prime }\right\}$, so that (31) can be rewritten in the following form

(32) $$\begin{array}{cc} E\;\left[{∥{\tilde{\psi }}_{k}^{\left(t\right)}∥}_{\sigma_{k}}^{2}\right]= & \;E\;\left[{∥{\tilde{\psi }}_{k-1}^{\left(t\right)}∥}_{F_{k}\sigma_{k}}^{2}\right]+g_{k}\sigma_{k} \end{array}$$

with $g_{k}≜\mu_{k}^{2}\sigma_{v,k}^{2}{\mathrm{vec}}^{T}\left\{H_{k}\right\}$. Observe that the expression (32) relates ${\tilde{\psi }}_{k}^{\left(t\right)}$ to ${\tilde{\psi }}_{k-1}^{\left(t\right)}$ (not ${\tilde{\psi }}_{k}^{\left(t-1\right)}$). To resolve this issue, the incremental topology along with the weighting matrices should be employed [19] as follows: first, by iterating (32) a set of K coupled equalities are obtained

(33) $$\begin{array}{cc} E\;\left[{∥{\tilde{\psi }}_{1}^{\left(t\right)}∥}_{\sigma_{1}}^{2}\right]= & \;E\;\left[{∥{\tilde{\psi }}_{K}^{\left(t-1\right)}∥}_{F_{1}\sigma_{1}}^{2}\right]+g_{1}\sigma_{1}\\ E\;\left[{∥{\tilde{\psi }}_{k}^{\left(t\right)}∥}_{\sigma_{2}}^{2}\right]= & \;E\;\left[{∥{\tilde{\psi }}_{1}^{\left(t\right)}∥}_{F_{2}\sigma_{2}}^{2}\right]+g_{2}\sigma_{2}\\ \; & \vdots \\ E\;\left[{∥{\tilde{\psi }}_{k-1}^{\left(t\right)}∥}_{\sigma_{k-1}}^{2}\right]= & \;E\;\left[{∥{\tilde{\psi }}_{k-2}^{\left(t\right)}∥}_{F_{k-1}\sigma_{k-1}}^{2}\right]+g_{k-1}\sigma_{k-1} \end{array}$$

(34) $$\begin{array}{cc} E\;\left[{∥{\tilde{\psi }}_{k}^{\left(t\right)}∥}_{\sigma_{k}}^{2}\right]= & \;E\;\left[{∥{\tilde{\psi }}_{k-1}^{\left(t\right)}∥}_{F_{k}\sigma_{k}}^{2}\right]+g_{k}\sigma_{k}\\ \; & \vdots \\ E\;\left[{∥{\tilde{\psi }}_{K}^{\left(t\right)}∥}_{\sigma_{K}}^{2}\right]= & \;E\;\left[{∥{\tilde{\psi }}_{K-1}^{\left(t\right)}∥}_{F_{K}\sigma_{K}}^{2}\right]+g_{K}\sigma_{K} \end{array}$$

In order to explain the flow of energy through the agents it is required to make connection between the free parameters σ and $\sigma^{\prime }$. If we choose the weighting vector $\sigma_{k-1}=F_{k}\sigma_{k}$ and combine (33) and (34) we get

(35) $$\begin{array}{cc} E\;\left[{∥{\tilde{\psi }}_{k}^{\left(t\right)}∥}_{\sigma_{k}}^{2}\right]= & \;E\;\left[{∥{\tilde{\psi }}_{k-1}^{\left(t\right)}∥}_{F_{k}\sigma_{k}^{\prime }}^{2}\right]+g_{k}\sigma_{k}\\ = & \;E\;\left[{∥{\tilde{\psi }}_{k-2}^{\left(t\right)}∥}_{F_{k-1}F_{k}\sigma_{k}}^{2}\right]+g_{k-1}F_{k}\sigma_{k}+g_{k}\sigma_{k} \end{array}$$

Iterating in this manner, a recursive expression is obtained which relates ${\tilde{\psi }}_{k-1}^{\left(t\right)}$ to ${\tilde{\psi }}_{k-1}^{\left(t-1\right)}$, namely

(36) $$\begin{array}{cc} E\;\left[{∥{\tilde{\psi }}_{k-1}^{\left(t\right)}∥}_{\sigma_{k-1}}^{2}\right]= & \;E\;\left[{∥{\tilde{\psi }}_{k-1}^{\left(t-1\right)}∥}_{F_{k}\ldots F_{K}F_{1}\ldots F_{k-1}\sigma_{k}}^{2}\right]\\ + & \;g_{k}F_{k+1}\ldots F_{K}F_{1}\ldots F_{k-1}\sigma_{k-1}\\ + & \;g_{k+1}F_{k+2}\ldots F_{K}F_{1}\ldots F_{k-1}\sigma_{k-1}.\\ \ldots & +g_{k-2}F_{k-1}\sigma_{k-1}+g_{k-1}\sigma_{k-1} \end{array}$$

By choosing $\sigma =q=\mathrm{vec}\left\{I_{L^{2}}\right\}$ and substituting it in (36), gives the following expression that explains how MSD of node *k* evolves over time:

(37) $$\begin{array}{cc} E\;\left[{∥{\tilde{\psi }}_{k-1}^{\left(t\right)}∥}_{\sigma_{k-1}}^{2}\right]= & \;E\;\left[{∥{\tilde{\psi }}_{k-1}^{\left(-1\right)}∥}_{{\left(\Gamma_{k-1,1}\right)}^{t}q}^{2}\right]\\ + & \;a_{k-1}\left(I_{L^{2}}+\ldots +{\left(\Gamma_{k-1,1}\right)}^{t-1}\right)q \end{array}$$

where $\Gamma_{k-1,\ell }\in R^{L^{2}\times L^{2}}$, the product of $F_{k}$ matrices for each node, and $a_{k-1}\in R^{1\times L^{2}}$ are defined by

(38) $$\begin{array}{cc} \Gamma_{k-1,\ell } & ≜F_{k+\ell -1}F_{k+1}\ldots F_{L}F_{1}\ldots F_{k-1},\;\ell =1,2,\ldots ,L \end{array}$$

and

(39) $$\begin{array}{cc} a_{k-1} & ≜g_{k}\Gamma_{k-1,2}+g_{k+1}\Gamma_{k-1,3}\ldots +g_{k-2}\Gamma_{k-1,K}+g_{k-1} \end{array}$$

Therefore, the theoretical expression for MSD of node *k* is given by the compact form

(40) $$\begin{array}{cc} E\;\left[{∥{\tilde{\psi }}_{k-1}^{\left(t\right)}∥}_{q}^{2}\right]= & \;E\;\left[{∥{\tilde{\psi }}_{k-1}^{\left(-1\right)}∥}_{\gamma_{t-1}}^{2}\right]+a_{k-1}b_{t-1} \end{array}$$

where the vectors $\gamma_{t-1}$ and $b_{t-1}$ are formulated by

$$\begin{array}{cc} \gamma_{t-1} & =\Gamma_{k-1,1}\gamma_{t-2},\;\gamma_{-1}=q\\ b_{t-1} & =b_{t-2}+\gamma_{t-1},\;b_{-1}=\mathrm{null}\;\mathrm{vector}\;\mathrm{of}\;\mathrm{size}\;L^{2}\times 1 \end{array}$$

Accordingly, the selection of $\sigma_{k-1}=r_{k}=\mathrm{vec}\left\{R_{u,k}\right\}$ leads to a an expression that explains how EMSE of node *k* evolves over time:

(41) $$\begin{array}{cc} E\;\left[{∥{\tilde{\psi }}_{k-1}^{\left(t\right)}∥}_{r_{k}}^{2}\right]= & \;E\;\left[{∥{\tilde{\psi }}_{k-1}^{\left(-1\right)}∥}_{\gamma_{t-1}^{\prime }}^{2}\right]+a_{k-1}b_{t-1}^{\prime } \end{array}$$

where the vectors $\gamma_{t-1}^{\prime }$ and $b_{t-1}^{\prime }$ are given, respectively, by

$$\begin{array}{cc} \gamma_{t-1}^{\prime } & =\Gamma_{k-1,1}\gamma_{t-2}^{\prime },\;\gamma_{-1}^{\prime }=r_{k}\\ b_{t-1}^{\prime } & =b_{t-2}^{\prime }+\gamma_{t-1}^{\prime },\;b_{-1}^{\prime }=\mathrm{null}\;\mathrm{vector}\;\mathrm{of}\;\mathrm{size}\;L^{2}\times 1 \end{array}$$

### 3.5. Steady-State Behavior

The variance relation (32) can also be used to evaluate the steady-state performance of CD-ILMS algorithm. Let $p_{k}≜{\tilde{\psi }}_{k}^{\left(\infty \right)}$. At steady-state, i.e., when t→∞, the variance relation (32) can be written as

(42) $$\begin{array}{cc} E\;\left[{∥p_{k}∥}_{\sigma_{k}}^{2}\right]= & \;E\;\left[{∥p_{k-1}∥}_{F_{k}\sigma_{k}}^{2}\right]+g_{k}\sigma_{k} \end{array}$$

Iterating the Equation (42) for an incremental network topology and selecting appropriate weighting vectors $\sigma_{k}$ for k=1,2,…,K, we obtain an equality only involving $p_{k-1}$, given by

(43) $$\begin{array}{cc} E\;\left[{∥p_{k-1}∥}_{\sigma_{k-1}}^{2}\right]= & \;E\;\left[{∥p_{k-1}∥}_{F_{k}\ldots F_{K}F_{1}\ldots F_{k-1}\sigma_{k}}^{2}\right]\\ + & \;g_{k}F_{k+1}\ldots F_{K}F_{1}\ldots F_{k-1}\sigma_{k-1}\\ + & \;g_{k+1}F_{k+2}\ldots F_{K}F_{1}\ldots F_{k-1}\sigma_{k-1}.\\ \ldots & +g_{k-2}F_{k-1}\sigma_{k-1}+g_{k-1}\sigma_{k-1} \end{array}$$

Using (38) and (39) to simplify the expression (43), we obtain

(44) $$\begin{array}{c} E\;\left[{∥p_{k}∥}_{\left(I_{L^{2}}-\Gamma_{k-1,1}\right)\sigma_{k-1}}^{2}\right]=a_{k-1}\sigma_{k-1} \end{array}$$

Equation (44) can be employed to evaluate the performance measures at node *k*, defined as follows:

(45) $$\begin{array}{ccc} \eta_{k} & ≜E\;\left[{∥p_{k-1}∥}_{q}^{2}\right],\;q=\mathrm{vec}\left\{I_{L^{2}}\right\}\; & \left(\mathrm{MSD}\right) \end{array}$$

(46) $$\begin{array}{ccc} \zeta_{k} & ≜E\;\left[{∥p_{k-1}∥}_{r_{k}}^{2}\right],\;r_{k}=\mathrm{vec}\left\{R_{u,k}\right\}\; & \left(\mathrm{EMSE}\right) \end{array}$$

Proper selection of the weighting vector $\sigma_{k-1}$ in (44) gives the required mean-square values. Selecting the weighting vector $\sigma_{k}$ as the solution of the linear equation $\left(I_{L^{2}}-\Gamma_{k-1,1}\right)\sigma_{k-1}=q$ or $\left(I_{L^{2}}-\Gamma_{k-1,1}\right)\sigma_{k-1}=r_{k}$, the desired MSD and EMSE at node *k* can be obtained as follows:

(47) $$\begin{array}{ccc} \eta_{k} & =a_{k-1}{\left(I_{L^{2}}-\Gamma_{k-1,1}\right)}^{-1}q,\; & \left(\mathrm{MSD}\right) \end{array}$$

(48) $$\begin{array}{ccc} \zeta_{k} & =a_{k-1}{\left(I_{L^{2}}-\Gamma_{k-1,1}\right)}^{-1}r_{k},\; & \left(\mathrm{EMSE}\right) \end{array}$$

The vector $a_{k-1}$ can be regarded as the effect of aggregating the transformed noise and the local data statistics from all the nodes in the incremental network topology and the matrix $\Gamma_{k-1,\ell }$ can be interpreted as the transition matrix for the weighting vector $\sigma_{k-1}$.

**Remark** **1.** *It should be noted that (47) and (48) are exactly the theoretical MSD and EMSE for full-update ILMS algorithm, which means that the CD-ILMS algorithm has the same steady-state error performance compared with the incremental LMS algorithm.*

## 4. Further Insight into the Proposed CD-ILMS

In order to gain more insight into the performance of CD-ILMS algorithm, in this section we compare the convergence rate and computational complexity of CD-ILMS algorithm with the regular full-update ILMS algorithm [19].

### 4.1. Convergence Rate

To make the analysis more tractable, we consider the following assumption.

**Assumption** **3.** *The missing probabilities $\left\{\pi_{k}\right\}$, the covariance matrices $\left\{R_{u,k}\right\}$, and the step-size $\left\{\mu_{k}\right\}$ are identical, i.e.,*

$$\pi_{k}=\pi ,\;\;\mu_{k}=\mu ,\;R_{u,k}=R_{u}\;\forall k$$

It can be seen from (13) that the evolution of weight-error vector for both CD-ILMS and ILMS algorithms are controlled by the modes of the following matrices

(49) $$\begin{array}{cc} Q_{\mathrm{coor}} & =\left(I_{L}-\mu_{k}\left(1-\pi_{k}\right)R_{u,k}\right),\;\forall k \end{array}$$

(50) $$\begin{array}{cc} Q_{\mathrm{full}} & =\left(I_{L}-\mu_{k}R_{u,k}\right),\;\forall k \end{array}$$

where the subscript ‘coor’ and ‘full’ denote, respectively, the stochastic coordinate-descent and full-update incremental implementation. Thus, under Assumption 3, we have

(51) $$\begin{array}{cc} Q_{\mathrm{coor}} & ={\left(I_{L}-\mu \left(1-\pi \right)R_{u}\right)}^{K} \end{array}$$

(52) $$\begin{array}{cc} Q_{\mathrm{full}} & ={\left(I_{L}-\mu R_{u}\right)}^{K} \end{array}$$

From (51) and (52), we find that the modes of convergence are given by

(53) $$\begin{array}{cc} \kappa_{\mathrm{coor},l} & ={\left\{{\left(1-\mu \left(1-\pi \right)\lambda_{l}\right)}^{K}\right\}}_{l=1,\ldots ,L} \end{array}$$

(54) $$\begin{array}{cc} \kappa_{\mathrm{full},l} & ={\left\{{\left(1-\mu \lambda_{l}\right)}^{K}\right\}}_{l=1,\ldots ,L} \end{array}$$

where $\lambda_{l}$ denotes the eigenvalues of $R_{u}$. Letting $\tau_{\mathrm{coor}}$ and $\tau_{\mathrm{full}}$ represent the largest number of time iterations that are required for the mean error vector, $E\;\left[{\tilde{\psi }}_{k}^{\left(t\right)}\right]$, to converge to zero. Then, it holds that

(55) $$\begin{array}{cc} \frac{\tau_{\mathrm{coor}}}{\tau_{\mathrm{full}}} & =\frac{\ln\left(\kappa_{\mathrm{full},l}\right)}{\ln\left(\kappa_{\mathrm{coor},l}\right)}\\ \; & =\frac{\ln\left({\left(1-\mu \lambda_{l}\right)}^{K}\right)}{\ln\left({\left(1-\mu \left(1-\pi \right)\lambda_{l}\right)}^{K}\right)}\\ \; & \overset{*}{\approx }\frac{-\mu \lambda_{l}}{-\mu \left(1-\pi \right)\lambda_{l}}=\frac{1}{1-\pi } \end{array}$$

where in step $\left(*\right)$ we considered $\ln\left(1-z\right)\approx -z$ as z→0.

**Remark** **2.** *Expression (55) illustrates the increase in the convergence time in the CD-ILMS algorithm. Since the convergence time is longer, the CD-ILMS algorithm may need more quantities to be exchanged through the network compared to the ILMS algorithm.*

### 4.2. Computational Complexity

We now provide a discussion on computational complexity of full-update ILMS and its coordinate-descent implementations. Let $\theta_{\mathrm{add}}\geq 0$ and $\theta_{\mathrm{mul}}\geq 0$ represent the number of real additions and multiplications, respectively, that are required for every entry of the gradient vector. In CD-ILMS algorithm, every node requires $\left(1-\pi \right)\left(\theta_{\mathrm{mul}}L+L\right)$ real multiplication and $\left(1-\pi \right)\left(\theta_{\mathrm{add}}L+L\right)$ real additions per iteration in adaptation step (10), while in the full implementation, every node requires $\theta_{\mathrm{mul}}L+L$ multiplications and $\theta_{\mathrm{add}}L+L$ additions per iteration. Moreover, Let $\beta_{\mathrm{coor}}$ and $\beta_{\mathrm{full}}$ denote the number of multiplications needed by the adaptation steps per iteration at each agent k in the CD-ILMS and full-update cases. Then,

(56) $$\begin{array}{cc} \beta_{\mathrm{full},k} & =\left(\theta_{\mathrm{mul}}+1\right)L \end{array}$$

(57) $$\begin{array}{cc} \beta_{\mathrm{coor},k} & =\beta_{\mathrm{full},k}-\left(\theta_{\mathrm{mul}}+1\right)L\pi \end{array}$$

If these algorithms require $\tau_{\mathrm{coor}}$ and $\tau_{\mathrm{full}}$ iterations to attain their steady-state values, then the total number of real multiplications at node *k*, represented by $\alpha_{\mathrm{coor},k}$ and $\alpha_{\mathrm{full},k}$, are obtained by

(58) $$\begin{array}{cc} \alpha_{\mathrm{coor},k} & =\beta_{\mathrm{coor},k}\times \tau_{\mathrm{coor}} \end{array}$$

(59) $$\begin{array}{cc} \alpha_{\mathrm{full},k} & =\beta_{\mathrm{full},k}\times \tau_{\mathrm{full}} \end{array}$$

so that employing (55) results in

(60) $$\begin{array}{cc} \frac{\alpha_{\mathrm{coor},k}}{\alpha_{\mathrm{full},k}} & =\frac{\beta_{\mathrm{coor},k}}{\beta_{\mathrm{full},k}}\times \frac{1}{1-\pi } \end{array}$$

Substituting (56) and (57) into the first item in (60), we obtain

(61) $$\begin{array}{cc} \frac{\beta_{\mathrm{coor},k}}{\beta_{\mathrm{full},k}} & =1-\pi \end{array}$$

Thus, from (60) and (61)

(62) $$\begin{array}{cc} \frac{\alpha_{\mathrm{coor},k}}{\alpha_{\mathrm{full},k}} & =1 \end{array}$$

**Remark** **3.** *It is obvious that, $\alpha_{\mathrm{coor},k}$ and $\alpha_{\mathrm{full},k}$ are essentially identical. This means that although the convergence of CD-ILMS algorithm to steady-state region takes longer, the overall computational complexity, i.e., savings in computation per iteration, does remain invariant. Generally speaking, in situations where the computational complexity per iteration require to be minimal, the coordinate-descent scheme is recommended. Moreover, the coordinate-descent scheme requires more iterations to achieve the same steady-state performance.*

**Remark** **4** **(Communication Costs).** *In coordinate-descent incremental and regular incremental schemes, each node k receives weight estimate, $\psi_{k,t}$, from its predecessor node k−1 in the cycle. In this manner the overall number of communications needed at each node per iteration is L. Moreover, the nodes also need to send their global estimate, $w_{t-1}$, thus the communications requirement per iteration per node is 2L.*

## 5. Simulation Results

**Figure 2:**
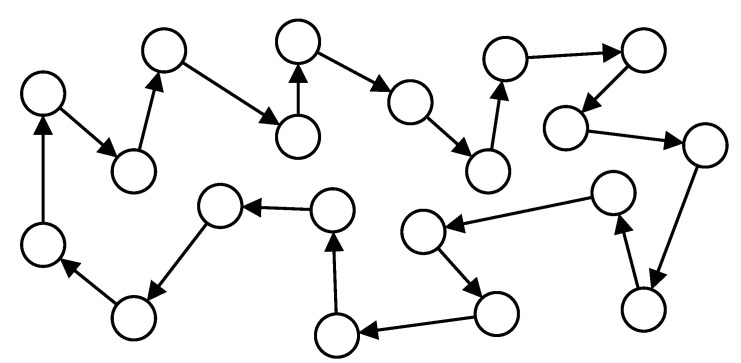
Network topology consisting of K=20 agents with ring topology.

**Figure 3:**
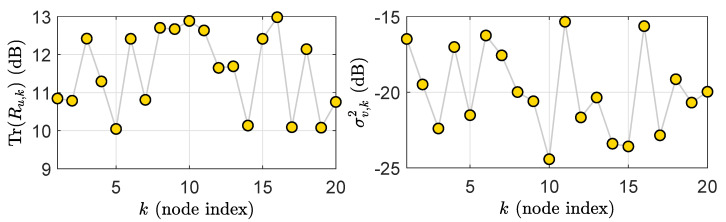
The network statistical settings: regressor power profile (**Left**) and observation noise power profile (**Right**).

This section provides some computer simulations to evaluate the performance of CD-ILMS algorithm and verify the theoretical analysis. We assume a distributed network composed of K=20 agents with ring topology (see Figure 2). The L=8 vector $w^{o}$ is generated randomly with $∥w^{o}{∥}^{2}=1$ and step-sizes are $\mu_{k}=0.02$. To obtain more accurate results, we conducted 100 independent simulations. Although the analysis depends on the independence assumptions, all simulations are accomplished by regressors with shift structure to deal with realistic scenarios. To this end, the regressors are generated by the following first-order auto-regressive model

$$\begin{array}{c} u_{k,t}\left(l\right)=z_{k}u_{k,t}\left(l-1\right)+\sqrt{1-z_{k}^{2}}h_{k,t}\left(l\right),\;1\leq l<L \end{array}$$

where the parameters $z_{k}\in \left(-1,\;1\right)$ and $h_{k,t}$ denotes a are zero-mean, unit-variance Gaussian sequences. For each node, the measurement noise $v_{k}\left(t\right)$ is a zero mean white Gaussian sequence with $\sigma_{v}^{2}\in \left(00.001,\;0.1\right)$. Figure 3 illustrates the network statistical settings.

**Figure 4:**
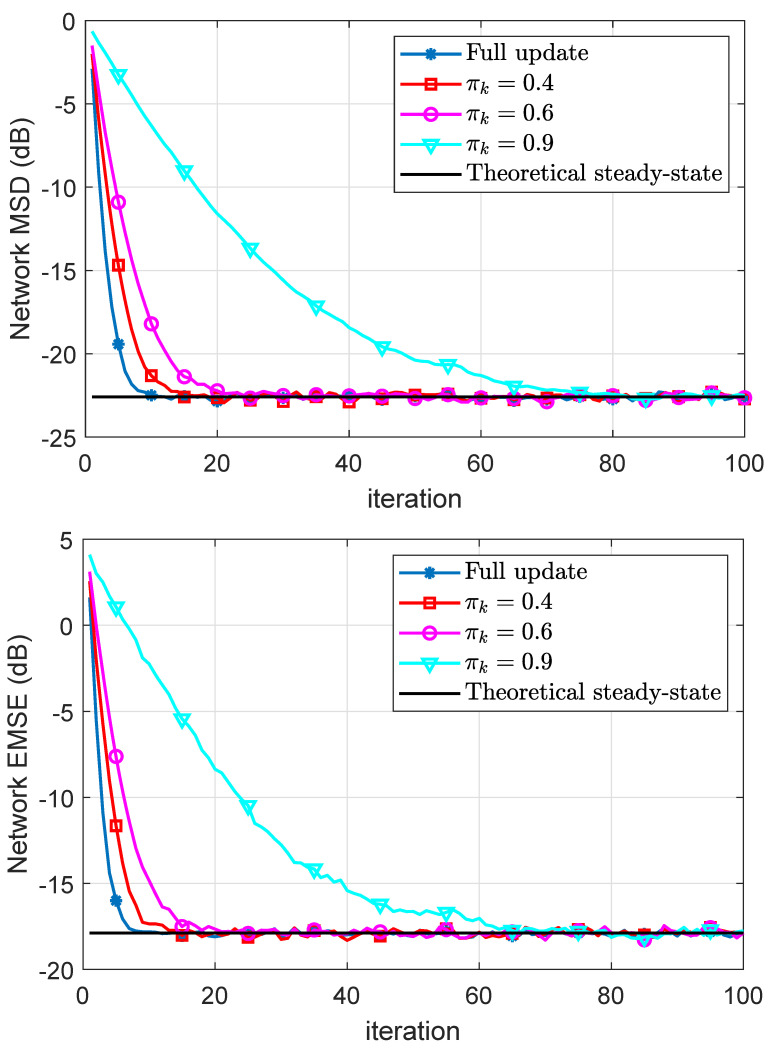
Simulated network MSD and EMSE curves and their theoretical results.

In Figure 4, we plot the experimental and theoretical MSD and EMSE learning curves using both full and partial updates for different values of $\pi_{k}\in \left\{0.4,0.6,0.9\right\}$. It can be observed that the CDILS algorithm has the same steady-state error performance compared with the ILMS algorithm. Moreover, as we expected, as $\pi_{k}\to 0$ the performance of the coordinate-descent approaches to that of the full-gradient incremental algorithm. Generally speaking, the speed of convergence reduces proportionally for coordinate-descent schemes as the missing probability is decreased.

**Figure 5:**
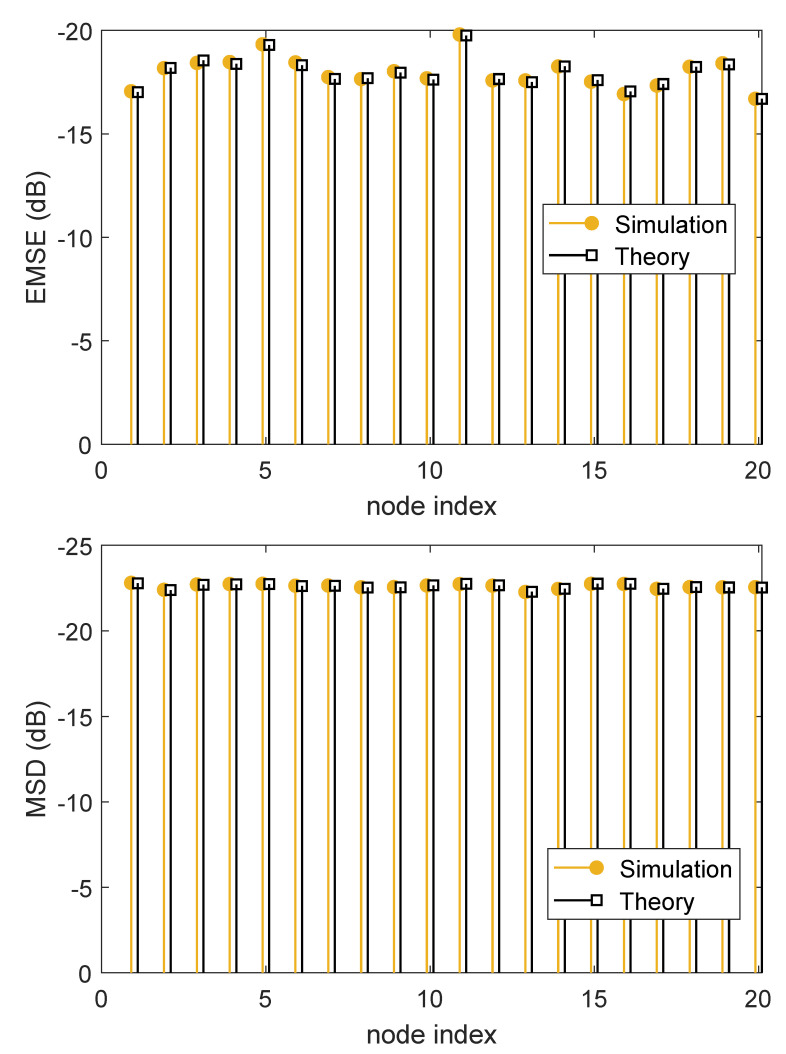
Theoretical and experimental steady-state MSD and EMSE at each node.

In Figure 5, we demonstrate the experimental and theoretical steady-state MSDs and EMSEs for $\pi_{k}=0.6$. It can be seen that, the calculated steady-state MSD and EMSE values using the theoretical expressions in (47) and (48) have a good agreement between with those obtained by the simulation results.

**Figure 6:**
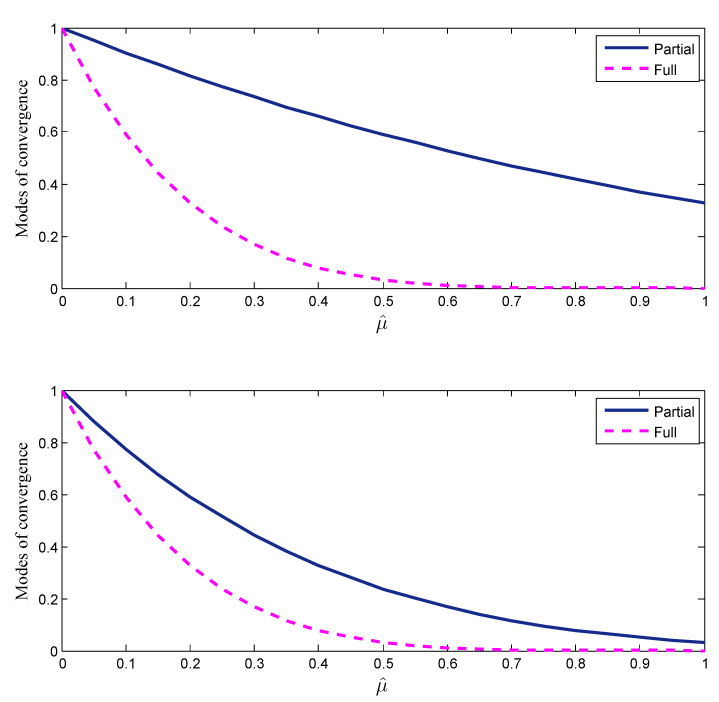
Modes of mean-convergence, for ILMS and CD-ILMS algorithms using K=10, π=0.8 (**top**) and π=0.5 (**bottom**).

Figure 6 shows the modes of convergence for the case $R_{u}=I_{L}$ and K=10, for $\pi_{k}\in \left\{0.5,0.8\right\}$, as a function of $\bar{\mu }=\mu \lambda$, both for ILMS and CD-ILMS algorithms. From Figure 6, it can be observed that, regardless of the values of step-size parameter, the full-update ILMS algorithm is always faster than the CD-ILMS algorithm.

## 6. Conclusions

In this paper, we have derived the CD-ILMS algorithm for adaptive estimation over incremental networks. Moreover, its detailed performance analysis based on the weighted energy-conservation approach under some assumptions and approximations has been discussed. More specifically, we have derived mean and mean-square stability conditions and theoretical expressions for steady-state and learning curves of MSD and EMSE. To gain further insight into the performance of CD-ILMS algorithm, its convergence and computational complexity have been compared with those of the full-update ILMS algorithm. It has been shown that the full-update ILMS algorithm and CD-ILMS algorithm provide the same steady-state performance, while full-update ILMS is always faster than the CD-ILMS algorithm. Finally, some numerical examples have been provided to support the theoretical derivations.
